# The Disruptive bEhavior manageMEnt ANd prevention in hospitalized patients using a behaviORal intervention team (DEMEANOR) study protocol: a pragmatic, cluster, crossover trial

**DOI:** 10.1186/s13063-020-04278-2

**Published:** 2020-05-24

**Authors:** Jay Morrison, Michele Hasselblad, Ruth Kleinpell, Reagan Buie, Deborah Ariosto, Erin Hardiman, Stephen W. Osborn, Christopher J. Lindsell, for the Vanderbilt Learning Healthcare System Investigators, Vanderbilt University

**Affiliations:** 1 Vanderbilt University Medical Center, Nashville, TN USA; 2 Vanderbilt University School of Nursing, 461 21st Ave, 407 GH, Nashville, TN 37240 USA; 3 Vanderbilt Institute for Clinical and Translational Research, Vanderbilt University Medical Center, Nashville, TN USA; 4 Department of Biostatistics and Vanderbilt Institute for Clinical and Translational Research, Nashville, TN USA

**Keywords:** Behavioral intervention team, Disruptive behavior, Hospitalized patients, Advanced practice registered nurse, Social work intervention

## Abstract

**Background:**

Disruptive behavior in hospitalized patients has become a priority area of safety concern for clinical staff, and also has consequences for patient management and hospital course. Proactive screening and intervention of patients with behavioral comorbidities has been reported to reduce disruptive behavior in some settings, but it has not been studied in a rigorous way.

**Methods:**

The Disruptive bEhavior manageMEnt ANd prevention in hospitalized patients using a behaviORal intervention team (DEMEANOR) study is a pragmatic, cluster, crossover trial that is being conducted. Each month, the behavioral intervention team, comprising a psychiatric-mental health advanced practice nurse and a clinical social worker, with psychiatrist consultation as needed, rotates between an adult medicine unit and a mixed cardiac unit at Vanderbilt University Medical Center in Nashville, TN, USA. The team proactively screens patients upon admission, utilizing a protocol which includes a comprehensive chart review and, if indicated, a brief interview, seeking to identify those patients who possess risk factors indicative of either a potential psychological barrier to their own clinical progress or a potential risk for exhibiting disruptive, aggressive, or self-injurious behavior during their hospitalization. Once identified, the team provides interventions aimed at mitigating these risks, educates and supports the patient care teams (nurses, physicians, and others), and assists non-psychiatric staff in the management of patients who require behavioral healthcare. Patients who are both admitted to and discharged from either unit are included in the study. Anticipated enrollment is approximately 1790 patients. The two primary outcomes are (1) a composite of objective measures related to the patients’ disruptive, threatening, or acting out behaviors, and (2) staff self-reported comfort with and confidence in their ability to manage patients exhibiting disruptive, threatening, or acting out behavior. Secondary outcomes include patient length of stay, patient attendant (sitter) use, and the unit nursing staff retention.

**Discussion:**

This ongoing trial will provide evidence on the real-world effectiveness of a proactive behavioral intervention to prevent disruptive, threatening, or acting out events in adult hospitalized patients.

**Trial registration:**

ClinicalTrials.gov: NCT03777241. Registered on 14 December 2018.

## Background

Patients who are hospitalized on non-behavioral health units who exhibit concurrent psychiatric comorbidities can be at risk for increased length of hospital stay due to the complexities associated with treatment, as well as behaviors that can be difficult to address by staff. Research has substantiated that 20–40% of patients hospitalized on general medical-surgical units have a psychiatric diagnosis [4, 7]. At our institution, 54% of all discharges on two medical-surgical units during fiscal year 2017 had a behavioral health diagnosis. These patients had a 25% longer mean length of stay (6.6 days vs 5.31 days without a behavioral health diagnosis) and 17% higher mean variable costs per discharge ($11,307 vs $9642), based on 104,843 total patient days.

At the same time, an internal “Behavioral Health Care Knowledge and Skills” assessment of 623 staff nurses identified that 72% reported patients’ behavior impacted their ability to provide care, 58% reported experiencing situational anxiety in caring for these patients, 56% reported caring for behavioral health patients daily to weekly (32% reported monthly), 50% reported feeling somewhat to very uncomfortable caring for these patients, and 44% feared for their personal safety as a result of patient disruptive behavior. These data demonstrate a large impact of patient behavior on the care that can be provided to them and on the satisfaction and morale of nursing staff.

Additionally our institutional data suggest nursing turnover rate is higher for those units with a higher burden of patients with behavioral comorbidities, and exit interviews suggest a link between turnover and the challenges of managing patients exhibiting disruptive behavior. Beyond turnover, there is considerable transfer from these units as nurses seek to improve their job satisfaction. Given the impact on patient care as well as on those providing care, identifying effective strategies for preventing and managing disruptive patient behavior has become a priority in promoting staff and patient safety.

Several academic medical centers have reported numerous benefits with the use of a behavioral intervention team (BIT), which provides proactive consultation and liaison psychiatric service. Observed outcomes included decreased length of stay and decreased use of constant companions (sitters) [4, 7, 8, 11, 13, 14]. However, most reports are anecdotal, and there is a lack of comparative effectiveness research supporting the impact of proactive measures to reduce disruptive behavior in hospitalized patients.

The aim of the present trial is to evaluate the impact of a BIT on one medical-surgical unit and a mixed cardiac care unit with a high proportion of patients with behavioral health comorbidities. We hypothesize that, when compared to usual unit staffing, the addition of a dedicated, trained BIT provides meaningful improvement in the prevention and management of disruptive behavior in the healthcare setting as well as improvement in staff perceptions of their ability to manage patients exhibiting disruptive, threatening, or acting out behavior.

## Methods

This manuscript was written in accordance with the Standard Protocol Items: Recommendations for Interventional Trials (SPIRIT) guidelines [2].

### Design

**Fig. 1:**
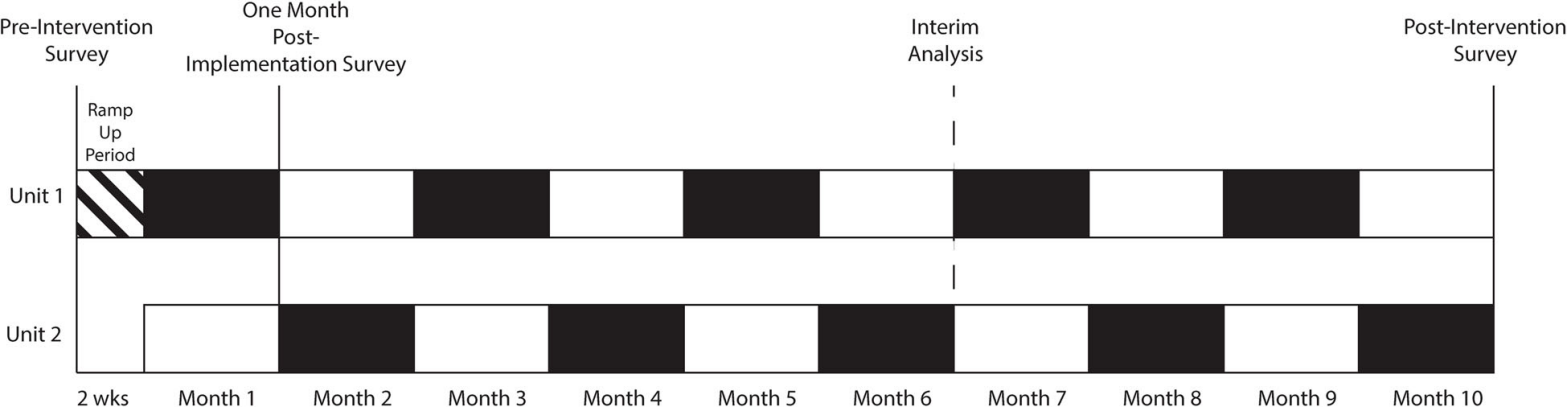
Depiction of the study implementation showing the ramp-up period and the crossover of the team between units. The timing of surveys and interim analysis is also shown

The Disruptive bEhavior manageMEnt ANd prevention in hospitalized patients using a behaviORal intervention (DEMEANOR) study is a single-center, pragmatic, cluster, crossover trial, testing the superiority of the BIT program to usual care, beginning 1 March 2019 on one medical-surgical unit and one mixed cardiac care clinical unit at Vanderbilt University Medical Center in Nashville, TN, USA. The BIT is deployed to each unit in alternating months, as shown in Fig. 1. Patients presenting to the unit during a month when the team is present contribute to the intervention arm, while those presenting to a unit when the team is not present contribute to the control arm.

### Study sites

The two study units are a 27-bed mixed cardiac medical-surgical unit and a 22-bed cardiac stepdown unit.

### Population

All adult (aged ≥18 years) patients admitted to either of the two units during the study period are eligible for the trial. To prevent contamination between study arms, patients must both be admitted to and discharged from the unit during the study month; patients present on a unit at the crossover will not be included in the analysis. In addition, all nursing staff working in these units during the study are included.

### Enrollment/randomization

Patients presenting to and discharged from participating units are enrolled automatically. As a cluster, crossover trial, randomization was limited to which unit the intervention would be deployed to first. Unit nursing staff were invited to participate in online surveys by e-mail, with reminders through staff meetings; their participation is voluntary.

### Intervention

The integration of the BIT into the unit is not itself a research intervention. Institutional leadership planned to deploy one team as a demonstration project, and to scale it if successful. This provided an opportunity for rigorous evaluation of the team’s effectiveness. The BIT, a multidisciplinary team comprising a psychiatric-mental health advanced practice nurse and a clinical social worker, with psychiatrist consultation as needed, is a modified version of an established model at Yale New Haven Hospital [14]. The team proactively screens patients upon admission to the unit, utilizing a protocol which includes a comprehensive chart review and, if indicated, a brief interview, seeking to identify those patients who possess risk factors indicative of either a potential psychological barrier to their own clinical progress or a potential risk for exhibiting disruptive, aggressive, or self-injurious behavior during their hospitalization. Once identified, the team provides interventions aimed at mitigating these risks through a variety of patient-specific interventions, including:

1. Psychiatric consultation and recommendations for symptom management
2. Behavioral plans of care for nurse/patient interaction
3. Psychosocial support and brief psychotherapeutic intervention
4. Curbside consultation for any member of the patient’s healthcare team
5. Patient advocacy and care coordination
6. Psychiatric-specific disposition support, including both inpatient and outpatient psychiatric services.

The team also provides education and support to the patient care teams (nurses, physicians, and others) and assists non-psychiatric staff in the management of patients who require behavioral healthcare.

All patients on a unit when the BIT is present will be eligible for BIT services, including those who are already admitted when the team crosses into the unit. During a month when the BIT is not present on a unit, the care and management of patients is not supported with proactive screening and management. Unit staff have access to all psychiatric or behavioral care routinely available in usual care. To ensure continuity of care, patients who continue to require psychiatric support at the time the BIT rotates off the unit will continue to receive care equivalent to the usual care condition. It is anticipated that monthly rotations will occur for 10 to 12 months.

All unit staff will be exposed to the intervention throughout the study, although after the first month of the study only those staff on the unit initially randomized to the intervention will have been exposed. This provides an opportunity for a comparison between exposed and unexposed staff. We note that patients present on the unit during the time of crossover are exposed to both the intervention and the control conditions. To avoid crossover effects and eliminate the logistical challenges of changing any behavioral care provider team during the admission, patients are excluded if they are present on a unit during crossover. This does lead to some possibility of contamination, since the BIT may be present in a unit during a control condition. However, the BIT comprises just two providers who were given strict instructions to avoid such contamination and who were also provided with training on how to defer questions or requests that would have resulted in contamination.

### Data collection

Data are being collected for patients and for unit staff. Patient information is documented as a component of usual clinical care, and those data needed for this study will be extracted from the electronic health record (EHR). Data include demographics, documentation of patient disruptive behavior and associated medication administration, and patient length of stay. Disruptive behavior was not previously specifically defined as part of routine clinical care, and documentation in the clinical chart was variable. As part of the study, new documentation fields were added to the EHR to enable description (and subsequent extraction) of specific patient behaviors. Disruptive patient behavior will be documented, including physical behaviors (hit, kick, slapped, shoved, spit at, thrown objects, grabbed, bitten, or attacked), verbal behaviors (threatening, bullying, harassing, name calling, blaming, insulting, yelling, cursing, intimidation), and any clinical interventions that are implemented to control any violent behavior.

Data will be extracted from the EHR by an automated query. The query will be validated by manual cross-check of a subset of cases. Data will be identified using an encounter number, allowing for merging of data for individual patients from different sources. The data files generated by manual query will be stored on secure servers and will be merged into a single dataset for analysis by the statistical team.

Anonymous surveys will be used to measure staff perceptions of their ability to manage disruptive patient behavior as well as their experience with the BIT. The behavioral health survey previously used at the organization is a 10-item instrument that assesses staff perceptions of workplace violence and safety (Table 1). The 10-item scale was adapted from prior work by Loucks et al. [9] and Rutledge et al. [12], who have validated the Behavioral Health Care Competency Survey. The survey was evaluated for content validity for the current purpose. For this study, we have adapted these instruments to be administered to nursing staff on the two participating units at three different time points: prior to the study, following the first 1-month intervention period, and after the study period has been completed (see Fig. 1). The surveys will be conducted electronically using Research Electronic Data Capture (REDCap) [5]. REDCap allows for surveys completed by the same individuals to be linked while maintaining the anonymity of respondents by preventing access to the linkage tables held securely within the REDCap application.

**Table 1:** Behavioral health survey to assess staff perceptions of workplace violence and safety

1. Have you encountered patients exhibiting disruptive, threatening, or acting out behavior?					
Yes					
No					
2. If yes, did their behavior in any way impact your ability to provide care?					
Yes					
No					
3. Please specify how care was impacted (select all that apply):					
Patient was non-compliant with care					
Took time away from care with other patients					
Disruptive to unit and other patients					
Patient was combative					
Other (please describe)					
4. In your current position, which of the following did you experience while caring for a patient exhibiting disruptive, threatening, or acting out behavior? (Select all that apply.)					
Physical (hit, kick, slapped, shoved, spit at, thrown objects, grabbed, bitten, or attacked)					
Verbal (threatening, bullying, harassing, name calling, blaming, insulting, yelling, cursing, intimidation)					
Elopement					
Central line manipulation related to substance abuse					
Suicidal ideation					
Self-injury					
Other (please describe)					
5. Did you experience any of the following because of caring for a patient exhibiting disruptive, threatening, or acting out behavior?					
	Never	Rarely	Sometimes	Often	Always
Fear for personal safety					
Fear for patient safety					
Situational anxiety					
A disruptive family member rather than a patient					
Decreased job satisfaction					
6. How comfortable are you with strategies in caring for patients exhibiting disruptive, threatening, or acting out behavior?					
Very comfortable					
Somewhat comfortable					
Neutral					
Somewhat uncomfortable					
Very uncomfortable					
7. What reporting structure do you utilize when you have experienced an event with a patient exhibiting disruptive, threatening, or acting out behavior? (Select all that apply.)					
Consult service					
Confidential online reporting system					
First report of injury					
Employee Assistance Program					
Involve the primary team					
Involve the one-up leader					
Do not report					
None of the above					
8. What has been the most beneficial training/support you have received in your current position at Vanderbilt to prepare you to provide care for patients exhibiting disruptive, threatening, or acting out behavior? (Please rank order.)					
Behavioral health and safety learning management education module					
De-escalation and trauma informed care education					
Unit-based education					
Psychiatric consult service					
9. What do you think would be helpful for ongoing bedside support in the care of patients exhibiting disruptive, threatening, or acting out behavior? (Please rank order.)					
Behavioral management techniques					
Safety restraints					
Therapeutic communication					
General education					
Other (please describe)					
10. On a scale of 0 to 10, where 0 is the lowest and 10 is the highest level, rate your level of confidence in caring for patients exhibiting disruptive, threatening, or acting out behavior on your unit.					

References: Adapted from Zicko et al. [15] and Loucks et al. [9]

### Primary outcomes

This study has two primary outcomes: (1) any documented interventions to manage disruptive, threatening, or acting out behavior, and (2) staff self-reported comfort with and ability to manage patients exhibiting disruptive, threatening, or acting out behavior. Documented evidence of intervention is defined as:

- Violence control
- Injurious behavior
- Administration, including as-needed administration, of the following medications for behavior management: quetiapine (Seroquel), alprazolam (Xanax), clonazepam (Klonopin), haloperidol (Haldol), lorazepam (Ativan), olanzapine (Zyprexa), risperidone (Risperdal), and ziprasidone (Geodon).

### Secondary outcomes

Secondary outcomes include the occurrence of each intervention separately, sitter use, use of physical or chemical restraints, patient length of stay, and unit nursing staff turnover (Table 2).

**Table 2:** Outcome measures and definitions

Outcome	Definition	How we measure
***Primary***		
**Violence control measures utilized, or patient injurious behaviors reported**	Composite of violence control nursing intervention; PRN medication administration of quetiapine, alprazolam, clonazepam, haloperidol, lorazepam, olanzapine, risperidone, or ziprasidone for behavior management; or nursing problem of violence risk or injurious behavior	Extraction of nursing documentation and medication administration from the electronic health record
**Nurse comfort and confidence in their ability to manage patients exhibiting disruptive, threatening, or acting out behavior**	Staff perceptions of workplace violence/safety, as well as perceived confidence, comfort, and most helpful training/support received	A behavioral health survey administered via Research Electronic Data Capture (REDCap) prior to intervention, 1 month after intervention began, and at the conclusion of the intervention
***Secondary***		
**Rate of unit nursing staff retention**	Amount of turnover (departure from the organization) experienced on each study unit while the study was conducted	Extracted from human resources records
**Patient length of stay**^**a**^	Number of days a patient spends in the hospital from the time of the order to admit to the time of discharge	Extracted from the electronic health record
**Individual components of the primary outcome:** **• Violence control intervention** **• PRN medications for behavior management** **• Nursing problem of violence risk or injurious behavior**	As above	As above
**Use of restraints**	Use of physical or chemical restraints to control patient behavior	Extracted from the electronic health record
**Use of sitters/patient attendants**	Use of sitters/patient attendants for patient observation	Extracted from the electronic health record

^a^Resource length of stay (RLOS): indicates hospital resource/bed utilization, irrespective of the change in patient status. The measure excludes the time that encounters spend as an “emergency department”-type patient but begins the instant a patient is given an order to admit as an inpatient and ends at discharge. This metric thus includes time a patient is in an emergency department bed, in the post-anesthesia care unit, or other location but has an inpatient status. The metric is viewed by average and median, and adjusted by the total Case Mix Index

### Power calculation

Approximately 17.5% of patients admitted to the two participating units required one or more of the behavioral interventions in the year prior to the study. To determine the number of months of data collection necessary to detect a reduction of 5% in the use of behavioral interventions between the intervention and control arms, we used the method described by Arnup et al. [1]. Based on patient length of stay and admit rates, we estimated between 89 and 105 patients would be eligible for inclusion each month (patients present on a unit at crossover will not be included in the analysis). Therefore, a cluster size of 90 was used. If the within-period correlation is 0.1 and the within-cluster within-period correlation is also about 0.1, we would achieve 80% power with 10 months of data collection (a total sample size of 1790 patients exposed to either the control or intervention arm). Since these estimates are based on assumptions, and it is possible that either a shorter or longer duration is needed, we have specified an interim analysis at 6 months to confirm the overall event rate and to estimate the observed correlations. No comparisons between groups will occur at this time; accrued data will be used only for sample size re-estimation.

### Data and safety monitoring and interim analysis

The study involves the collection of data to compare patients who are or who are not exposed to a clinical care practice: the behavioral intervention team (BIT). As such, there is no research intervention. The principal investigator (PI) and co-PIs, in conjunction with the clinical nursing unit leadership, clinical nurse leaders, and clinical nurse educators, will monitor for any potential impact of the study that may cause untoward impact on patients or staff on an ongoing basis throughout the study duration. If any untoward impact is observed, it will be reported to the study investigators and the Institutional Review Board (IRB).

At 6 months, the data will be reviewed to determine how many additional months of data collection will be required to have sufficient power to detect the 5% reduction in behavioral outcomes that is being designated as minimally important. Based on power calculations, 6 months is the minimum period of time for which the study can run to find this difference. At 6 months, there will be sufficient information to more precisely estimate the magnitude of the within-cluster and within-cluster within-period correlation to update the sample size estimate. Formal stopping rules for safety, efficacy, or futility have not been designated.

### Statistical analysis principles

There are two main analyses. The first will compare outcomes between patients exposed to the BIT and patients not exposed to the BIT. The outcomes are quantitative variables that are either binary or ordinal in nature. Comparisons will use logistic regression or proportional odds models, adjusted for covariates. The models will include cluster (unit) as a random effect and will also include period, including the following patient characteristics: age, sex, race, comorbidities, reason for admission, and psychiatric diagnoses.

The second analysis will compare staff perceptions between those exposed to the behavioral intervention and those not exposed to the behavioral intervention during the first month, and will also compare perceptions before exposure to the behavioral intervention with perceptions after the study concludes. We will compare categorical variables using chi-square tests, and we will compare continuous variables using paired or independent samples *t* tests as appropriate. For continuous data that are non-normally distributed, and for ordinal variables, we will use the Mann-Whitney *U* test or the Wilcoxon test as appropriate.

While our initial approach is a simple comparison, this ignores the repeated measurements. Since staff on the unit can leave and new staff can join the unit, we will use a mixed-effects model to analyze differences between before the study, at 1 month after the intervention began, and on completion of the study. Respondents will be included as a random effect. Linear, logistic, or proportional odds models will be used as appropriate for the outcome. Finally, we may model the survey outcomes using similar regression techniques to explore factors associated with changes in staff perceptions.

### Treatment of outliers

Our evaluation of whether or not an individual patient experiences an event reduces the impact of outliers on the primary outcome. For survey results, the results are constrained to the available options, again limiting outliers. Therefore, any statistical modeling will not evaluate the impact of outliers. There is the possibility that there are outliers for variables such as length of stay or for counts of behavioral problems. These are expected and will be reported. These outcomes will generally be compared between groups or modeled using non-parametric approaches, which are robust to the presence of such outliers.

### Presentation of the results

After completion of enrollment and data analysis, the results of the trial will be communicated to the public through manuscript publication and submission of the results to the ClinicalTrials.gov database. Submission for publication will include public access to the full study protocol and statistical code. Authorship will be based on the International Committee of Medical Journal Editors guidelines [6], and professional writers will not be used.

The flow of patients through the study will be presented in a flow diagram (Fig. 1). Baseline characteristics will be presented by treatment group, as shown in Table 2. The percent of patient injurious behaviors reported will be determined by extracting reports of biting, kicking, throwing, etc., from the EHR. The percentage of violence control measures used will be determined by extracting medications, restraints, and sitters ordered from the EHR.

A composite of all documented indications of disruptive patient behavior, including violence control nursing interventions, as-needed (PRN [*pro re nata*]) medication administration for behavior management, nursing problem of violence risk, or any injurious behavior, will be extracted from the EHR. Each component of the primary outcome will also be reported separately, along with documented physical restraints for disruptive patient behavior.

The percentage change in nurse comfort and confidence in their ability to manage patients exhibiting disruptive, threatening, or acting out behavior; in perceptions of workplace violence/safety; and in perceived confidence, comfort, and most helpful training/support received will be determined by comparing surveys conducted pre-implementation, 1 month after the intervention began, and post-implementation.

The rate of unit nursing staff retention will be determined by extracting rates from internal human resources records. Patient length of stay will be determined by extracting data from the EHR.

## Discussion

Upon completion, the DEMEANOR study will provide the most comprehensive data to date on the impact of a proactive behavioral intervention on disruptive, threatening, or acting out events in adult hospitalized patients.

Several potential threats to the validity of the trial exist. As the primary and secondary variables are being collected from documentation in the EHR, the potential for incomplete data exists. Additional limitations in using EHR data in clinical research include ensuring data security and privacy and overcoming challenges associated with linking clinical data from diverse systems [3]. The use of sitters or virtual sitters and restraints is dependent on a variety of patient care conditions, and verifying those that pertain to disruptive patient behavior will be dependent on clinical documentation or specific ordering directives.

There is a possibility that patients hospitalized during the study period who did not receive a specific consult from the behavioral intervention team (BIT) nonetheless benefited from the presence of the team. The study design does not allow a conclusive differentiation between a spillover effect on the units where the BIT is implemented and a possible secular trend from other unmeasured influences on the primary or secondary outcomes. Consults on the control unit during the study period will be monitored to assess for the potential for an increase due to staff awareness and exposure to the BIT during the crossover periods. Potential fluctuations in patient census during the trial and also the number of patients presenting with behavioral comorbidities or those exhibiting disruptive behavior may also impact the study results.

Nursing response rates to the pre-study, 1 month, and post-implementation surveys may impact the ability to evaluate change in perceptions of their ability to manage patients exhibiting disruptive, threatening, or acting out behavior. Also, nursing retention rates are dependent on a number of factors, including career opportunities which may not be related to nursing job dissatisfaction or to encountering patients with disruptive behavior.

The DEMEANOR study is a pragmatic trial which provides the opportunity to evaluate the effectiveness of an intervention in real-life routine practice conditions [10]. It also allows us to consider the infrastructure and costs associated with implementing this type of program so that the results can be placed in this context. Inherent in this design however is the potential for a lack of external validity. Additionally, with a single-center trial, there is limited ability to generalize the study results.

## Trial status

The DEMEANOR study is an ongoing, single-center, pragmatic, crossover trial that will provide comprehensive information on the impact of a proactive behavioral intervention on disruptive, threatening, or acting out events in adult patients. The protocol is the current version (number one). Patient enrollment began on 1 March 2019, and enrollment ended 30 December 2019. Post implementation nurse surveys were completed between January and February 2020.

## Data Availability

On completion of the study, investigators external to the research team may request to collaborate on secondary analyses. With appropriate IRB approval and data use agreements in place, de-identified datasets may be released. All statistical code will be made publicly available with the analysis. The investigators plan to publish trial results without assistance from outside professional writers. The investigators have no publication restrictions.
